# Morphogenetic Study on the Maturation of Osteoblastic Cell as Induced by Inorganic Polyphosphate

**DOI:** 10.1371/journal.pone.0086834

**Published:** 2014-02-03

**Authors:** Kaori Tsutsumi, Nagahito Saito, Yumi Kawazoe, Hong-Kean Ooi, Toshikazu Shiba

**Affiliations:** 1 Department of Biological Sciences and Engineering, Faculty of Health Sciences, Hokkaido University, Kita-ku, Sapporo, Japan; 2 Department of Internal Medicine, Gastroenterology & Hematology Section, Hokkaido University Graduate School of Medicine, Kita-ku, Sapporo, Japan; 3 Regenetiss Inc., Kunitachi, Tokyo, Japan; 4 Department of Veterinary Medicine, Azabu University, Chuo-ku, Sagamihara-Shi, Kanagawa, Japan; 5 Laboratory for Polyphosphate Research, The Kitasato Institute, Kitasato Institute for Life Sciences, Kitasato University, Minato-ku, Tokyo, Japan; Brigham and Women's Hospital, Harvard Medical School, United States of America

## Abstract

Since inorganic polyphosphates [poly(P)] have an activity to induce bone differenciation in vitro and in vivo, we examined an effect of poly(P) on organelle by light microscopy and electron microscopy in Murine MC3T3-E1 osteoblastic cells. The MC3T3-E1 cells were ultrastructurally observed to possess morphological characteristics of osteoblasts. Cells cultured with poly(P) were strongly stained with an anti-collagen type I antibody but not in those cultured without poly(P). Ultrastructural analysis of cells cultured with poly(P) revealed a well-developed Golgi apparatus, swollen and elongated rough endoplasmic reticulum, large mitochondria and many coated pits. Since MC3T3-E1 cells can be transformed from a resting phase to an active blastic cell phase after supplementation with poly(P), it implies that poly(P) can be an effective material for bone regeneration.

## Introduction

Artificial regeneration of certain organs has been studied extensively in recent years with the use of immature cells and stem cells [1], [2], [3]. Although the use of induced pluripotent stem cells is expected to give impetus to regenerative medicine, it is still difficult to apply the technology in a clinical setting [4]. In orthopedic surgery, transplantation of osteoinductive mesenchymal stem cells (MSC) and osteoblasts for bone regeneration in patients with osteoporosis or bone deficiency, has been proposed [5], [6], [7]. Although the success of transplantation using blastic cells to yield mature and complete bone tissues is still obscure [1], [8], several investigations on the *in vitro* use of animal or human cells for bone regeneration have been reported [1], [9]. Furthermore, it was observed that mineralization of osteoblastic cells can be induced by treating the cells with β-glycerophosphate/ascorbic acid, dexamethasone or 1,25-dihydroxyvitamin D3 [9], [10], [11], [12].

MC3T3-E1 cell line was established from mouse calvaria and is thought to possess osteoblastic cell characteristics such as alkaline phosphatase activity and intracellularly deposited minerals [10]. However, its cellular ultrastructure has not yet been studied. Inorganic polyphosphate [poly(P)] is a linear polymer containing tens to hundreds of orthophosphate residues linked by high-energy phosphoanhydride bonds. In mammals, they are located in erythrocyte as well as in the cells of the brain, heart, lung and liver [13], [14], [15], [16]. It was previously shown that poly(P) promotes intracellular calcification and modulates the mitogenic activity of the fibroblast growth factor [17]. Besides inducing the alkaline phosphatase activity in MC3T3-E1 cells, poly(P) also up-regulates the osteopontin and osteocalcin genes [12]. From the findings of the cell culture systems, poly(P) is thought to play an important role in the maturation of bone-related immature cells such as MC3T3-E1 cells, which might lead to the construction of bone tissues by osteoblast transformation. Although poly(P) has been reported to function as a regulatory factor for gene expression [18], [19], its physiological activity in mammalian cell still need to be elucidated. Since poly(P) has been identified as a biopolymer in mammalian cell, and is also being used as a food-additive as well as in cosmetic material, it is deemed safe to be used in clinical medicine.

Using light and electron microscopy, we reported in this study that characteristic changes of organelle structure in MC3T3-E1 after culturing with poly(P) is related to bone differentiation.

## Materials and Methods

### Cell culture

MC3T3-E1 cells (Riken CELL BANK, Tokyo) were cultured in α-minimum essential medium (α-MEM, Gibco Co., Tokyo) supplemented with 10% fetal bovine serum (FBS, Gibco Co., Tokyo) and 50 µg/ml of Kanamycin (Wako Pure Chemical Industries Ltd., Tokyo) at 37°C in 5% CO2. The volume of medium used for the cell culture was 0.6 ml/well in cluster plate, 0.1 ml in Biocoat Insert (Becton Dickinson Co., U.S.A.) and 5 ml in a culture dish, respectively. The cells were seeded at a density of 2×10^4^ cells/cm^2^ and grown for 3 days until they reached confluence. The culture medium was then replaced with α-MEM containing 0.5% FBS and the cells were further cultured for 48 hours. To feed the cells with poly(P), the culture fluid was replaced with α-MEM supplemented with 0.5% FBS containing 1 mM poly(P) (sodium phosphate glass type 65, average chain length of 65, Sigma Chemicals Co., Tokyo) and cultured for the next 17 days (poly(P)-treated cells). The culture medium was changed every 3 days. As control, sodium phosphate buffer (pH 6.9) was used instead of poly(P).

### Phase contrast microscopy and immunofluorescence studies

The MC3T3-E1 cells were observed daily during culture using phase contrast light microscopy (Leica, Tokyo). On each day of the culture, a portion of the cells was fixed in 4% paraformaldehyde in 0.1 M Tris buffered saline (TBS, pH 7.4) for 15 min at 20°C, followed by washing with 0.1 M TBS containing 1 mM calcium (TBS-Ca). The fixed cells were then treated with 100% methanol for 30 min at −20°C and blocked with 5% skim milk in TBS-Ca. Immunofluorescent test on the cells was carried out using a rabbit-polyclonal antibody against collagen type I (diluted 1∶150, Chemicon International Inc., U.S.A.) and against osteopontin (diluted 1∶150, LSL co., Ltd., Japan) to observe for signs of early stage of calcification. Briefly, the cells were incubated with the primary antibody at room temperature for 1 hr and reacted with FITC-conjugated anti-rabbit IgG as a secondary antibody after washing three times with TBS-Ca for the observation of collagen type I protein. For the detection of osteopontin, in place of FITC-conjugated anti-rabbit IgG,, DAPI (100 ng/ml of 4′6-diamino-2-phenylindole dihydrochloride, Wako Pure Chemical Industries Ltd., Tokyo)-conjugated anti-rabbit IgG was used instead. Immunofluorescence in the cells was observed in a medium containing 0.1% p-phenylenediamine in PBS with glycerol (PPDA, Wako Pure Chemical Industries Ltd., Tokyo) under a fluorescence microscope (Leica, Tokyo).

### von Kossa staining

Cells were fixed with phosphate-buffered formalin for 20 minutes, washed once with distilled water and treated with 5% silver nitrate solution under ultraviolet irradiation (wavelength peak at 254 nm) for 2 hours. The samples were then washed with distilled water, treated with 5% thiosulfate for 10 min and subsequently dehydrated in an ethanol series for observation.

### Electron microscopy

On each of the day during culture, both the control and poly(P)-treated cells seeded in Biocoat Control Insert Microm (Becton Dickinson Ltd., U.S.A.), were fixed in 2% glutaraldehyde in 0.05 M cacodylate buffer (pH 7.4) for 30 min and then post-fixed in 2% osmium tetroxide at 4°C for 1 hour. The cells were dehydrated with increasing concentrations of ethanol and subsequently cleared in acetone. Finally, the cells were embedded in Epok 812 (Okenshoji Co., Tokyo) and kept at 70°C for 72 hours. Ultrathin sections made with ultra-microtome were stained with both lead citrate and uranyl acetate for observation under a transmission electron microscope (Hitachi H-800, Tokyo).

## Results

### Morphological changes observed by phase contrast light microscopy

**Figure 1:**
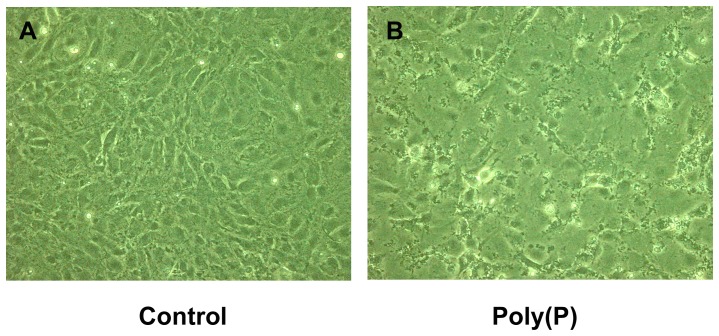
Morphological change in poly(P)-treated cells. Phase contrast light microscopy. (Original magnification, ×200) A. MC3T3-E1 cells on 3rd day of culture. Cytoplasm extended like a rugby ball. B. MC3T3-E1 cells treated with poly(P) on the 3rd day of culture. Cells are thicker and the cytoplasm irregularly extended with vacuoles.

Phase contrast light microscopy clearly revealed morphological differences between the control and poly(P)-treated cells on the 3rd day of culture; the control cells were uniformly thin with smooth extended cytoplasm throughout the observation period, whereas the poly(P)-treated cells gradually became difficult to focus with the passage of time in culture, due to increase in thickness of the cells. Indeed, it was difficult to observe numerous poly(P)-treated cells using a fixed focus. Many small vacuoles were seen adhering intra-cytoplasmically to the cell membranes of the poly(P)-treated cells (Figure 1).

### Localization of collagen type I and osteopontin

**Figure 2:**
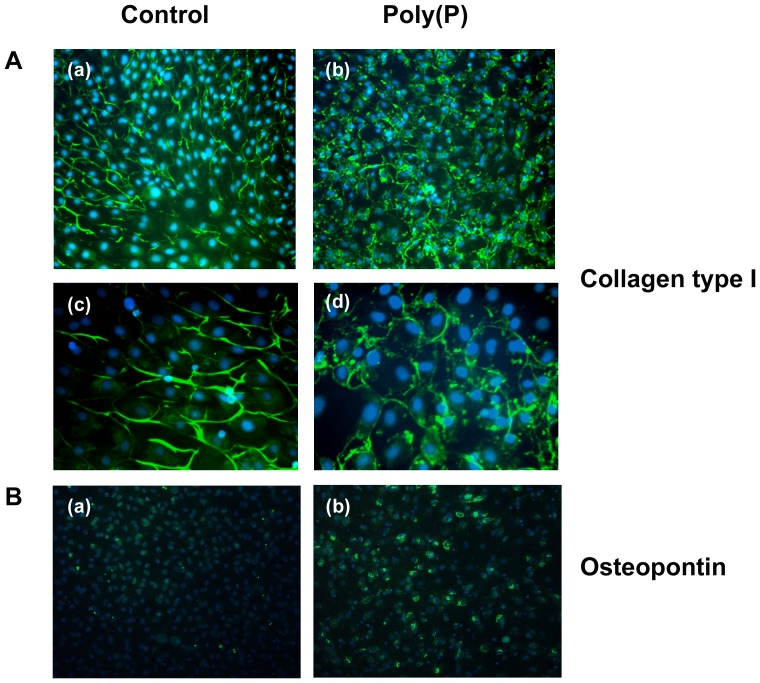
Effect of poly(P) on localization of collagen type I and osteopontin. A. Immunofluorescence using anti-collagen type I antibody on the 9th day of culture. (Original magnification, ×200 in a and b, ×400 in c and d) (a) Control cells. Peripheral area of the cytoplasm weakly positive. (b) Poly(P)-treated cells. Immunofluorescence intensity stronger than that of the control. Positive granular structures along the cytoplasm can be seen. (c) Higher magnification of Figure 2a. (d) Higher magnification of Figure 2b. B. Immunofluorescence using anti-osteopontin antibody. (Original magnification, ×200) (a) Control cells. Reaction is weakly positive. (b) Poly(P)-treated cells. Area around the nucleus shows strong positive reaction.

The poly(P)-treated cells also showed stronger immunofluorescence for collagen type I protein stain than control cells. This positive reaction in the poly(P)-treated cells with anti-collagen type I antibody was seen as granules localized to the peripheral area, which seemed to coincide with the location of vacuoles observed by phase contrast microscopy (Figure 2A). Furthermore, stronger fluorescence was observed in the poly(P)-treated cells than in control cells when stained with DAPI-conjugated antibody against anti-osteopontin antibody on the 9th day of culture (Figure 2B).

### Induction of cell calcification by poly(P)

**Figure 3:**
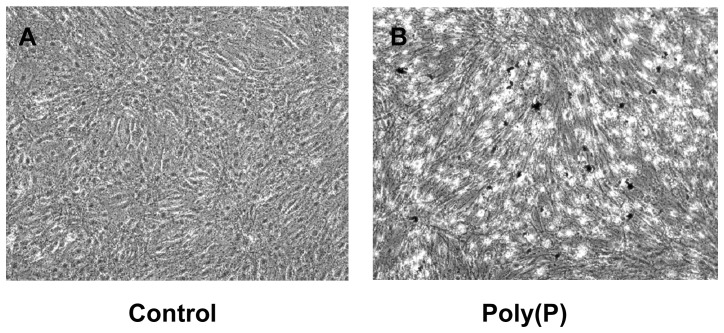
Effect of poly(P) on caltification in MC3T3-E1 cells. Von Kossa staining of MC3T3-E1 cells was carried out on the 38th day of culture after treatment with A. Control cells. B. Poly(P)-treated cells. (Original magnification, ×100).

On the 38th day of the culture, many nodules were observed by von Kossa staining in the poly(P)-treated cells, but no such structure was observed in the control cells (Figure 3).

### Morphological changes observed by electron microscopy

**Figure 4:**
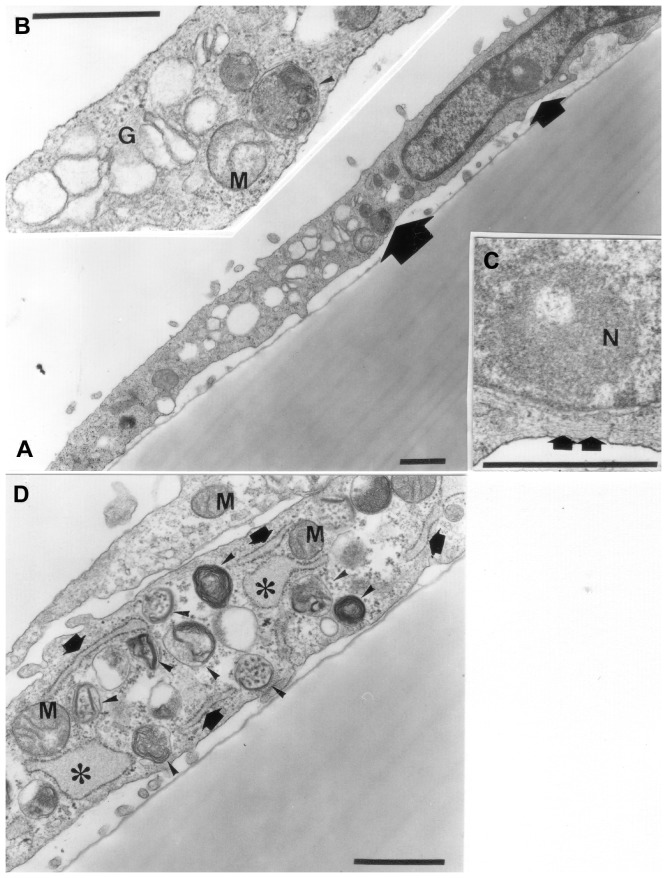
Electron micrograph of control cells on the 3rd day of culture. A. Slightly condensed chromatin in the nucleus. Nucleolus clearly demarcated. B. Higher magnification of area shown by large arrow in Figure 4A. Rounded mitochondria (M) and well-developed Golgi apparatus (G) can be seen. A granule (arrowhead) containing dense material, small granular structure and filamentous structure can also be seen. C. Higher magnification of area shown by small arrow in Figure 4A. Nucleolus (N) and bundle of microfilaments (double arrow) can be seen. D. Cytoplasm of control cells. Granules containing several myelin-like structures (arrowheads) visible. Rounded mitochondria (M) and elongated rRERs (arrow) can be seen. Several RERs (asterisks) swollen at one end. (Bar = 0.5 µm).

**Figure 5:**
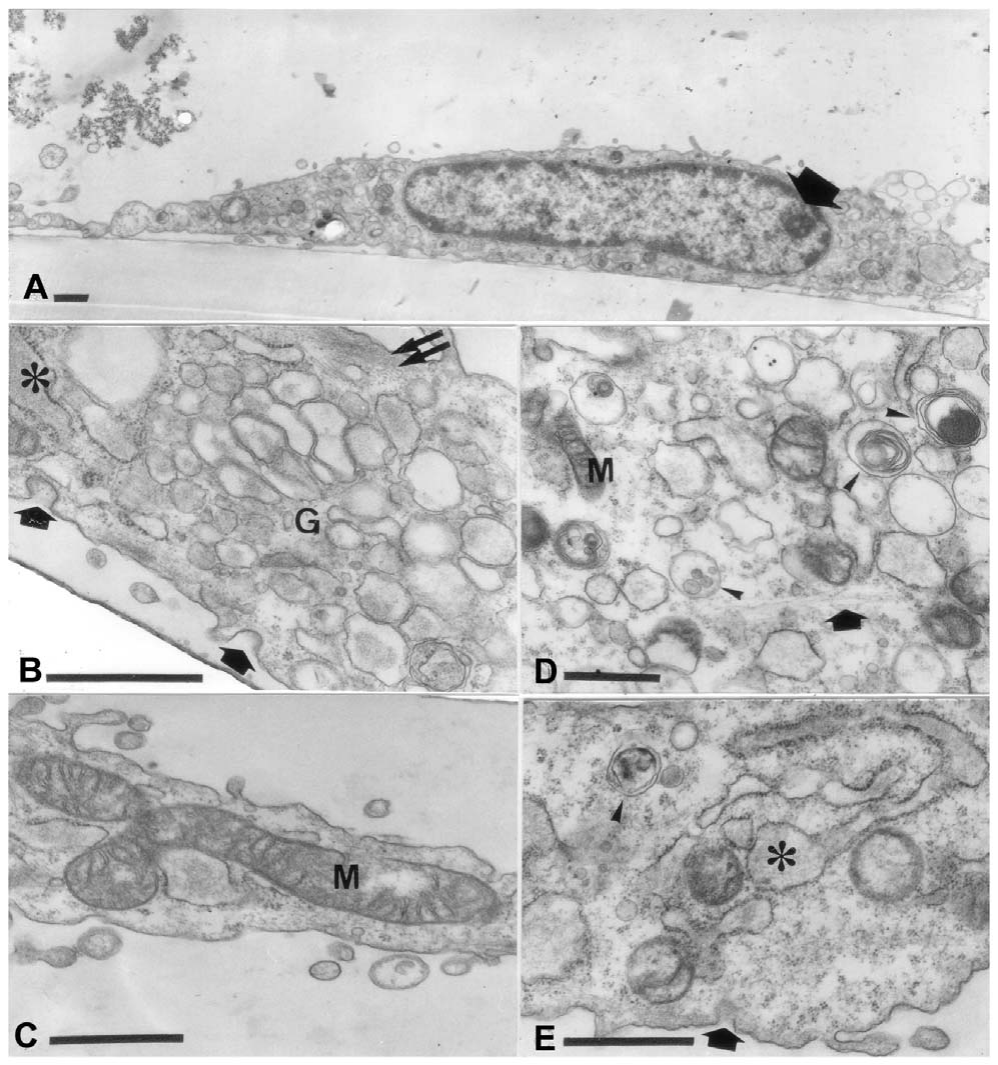
Electron micrograph of poly(P) cells on the 3rd day of culture. A. Nucleolus (arrows) is smaller. Vacuoles seen outside of cytoplasm (arrowhead). B. A well-developed Golgi apparatus (G) can be seen. Coated pits (arrows) and swollen RER (asterisk) are frequently observed. A bundle of microfilaments (double arrow) can also be seen. C. Mitochondria (M) large and elongated. D. Low density cytoplasm with many vacuoles containing small granules and myelin-like structures (arrowhead). E. RER well developed, elongated and swollen at one end (asterisk). A coated pit (arrow) and a granule-like structure with an amorphous material (arrowhead) can be seen. (Bar = 0.5 µm).

In control cells, the nucleus had a clearly demarcated nucleolus, and the chromatin was slightly condensed along the inside of the nuclear membrane. The cytoplasm consist of a rather dense matrix, with many free ribosomes, a well-developed Golgi apparatus with small vacuoles, elongated rough endoplasmic reticula (RERs) with a small bulge at one end, small rounded mitochondria with a dark matrix and dense cristae, small vacuoles containing myelin-like, amorphous or indefinite structures, microfilamentous structures near the nuclear membrane and a few coated pits (Figure 4). On the other hand, the poly(P)-treated cells, possessed more cell organelles than control cells. The nucleolus was smaller and displayed an indistinct border within the nucleus. In the cytoplasm of the poly(P)-treated cell, a more-developed Golgi apparatus with larger vacuoles, bigger elongated RERs with a swollen end, greatly lengthened mitochondria, much more vacuoles with irregular myelin-like, amorphous or indefinite structures, bundles of microfilaments, many more coated pits, and a lump of small vacuoles outside the cytoplasmic membrane were observed (Figure 5).

## Discussion

Our present study showed that treatment of MC3T3-E1 cells with poly(P) resulted in intracellular morphological changes. Poly(P) is found abundantly in normal human osteoblasts, in which it regulates the expression of the fibroblast growth factors [14], [16], [17], [20]. In our previous study, we observed enhanced alkaline phosphatase activity, and expression of osteopontin and osteocalcin genes in MC3T3-E1 cells, after treatment with inorganic polyphosphate [12]. These intracellular activities were thought to be due to the production of collagen type I protein in the cells and intracytoplasmic calcification induced by poly(P) during the early active stage.

In our present study, the appearance of osteopontin, as evidenced by the immunofluorescence study and the presence of many nodules as demonstrated by von Kossa staining suggest that treatment of the cells with poly(P) induced cellular calcification. Production of collagen type I protein in immature bone cells has been reported as a hallmark when the process of creating new bone cell is initiated, that is, osteoblastic cells going through the maturation phase [16], [21]. Dissolved poly(P) in cells may be a source of phosphates that acts as a positive signal for osteopontin expression [12], [21]. In this context, our demonstration of collagen type I protein by immunofluorescence, observation of small vesicles in the peripheral area of the cell by phase contrast microscopy and the showing of vacuoles in the vicinity of the cytoplasmic membrane by electron microscopy in the poly(P)-treated cells, seems to be inter-related to each other. However, further experiments, such as electron microscopic immunostaining using anti-collagen type I antibody, are needed to determine whether the vesicles observed by phase contrast microscopy and the vacuoles seen in electron microscopy coincide with the granular staining of collagen type I protein by immunofluorescence. In addition, electron micrographs also showed that the MC3T3-E1 control cells exhibited the morphological characteristics of osteoblasts which has been reported previously [10].

Since the RER and mitochondria are associated to calcium homeostasis and calcium signaling [21], [22], [23], [24], the presence of numerous coated pits in the poly(P)-treated cells is thought to be related to the uptake of various extracellular substrates for the activation of calcium homeostasis or signaling. In addition to the promotion of cellular uptake of extracellular substrates as indicated by the appearance of the coated pits, the development of RER and Golgi apparatus showed the activation of generation of the intracellular glycoprotein [25]. Furthermore, the morphological changes induced by poly(P) treatment in MC3T3-E1 cells suggest that these cells had entered the maturation or active stage of protein synthesis and calcification, which will subsequently lead to bone formation. Although the mitochondrial membrane contains apoptosis-related genes such as bcl-2 and poly(P) has been reported to be associated with apoptosis induction in plasma cell [26], [27], [28], our electron micrographs of the poly(P)-treated cells showed no evidence of apoptosis such as aggregation of nuclear chromatin, formation of apoptotic bodies, decrease in cell volume and budding formation, even on the 15th day of culture. On the other hand, in the poly(P)-treated cells, the nucleolus and chromatin were agglutinated in the nucleus and the more-developed Golgi apparatus, bigger elongated RERs and many vacuoles containing small granules indicated that glycoproteins were produced in the cytoplasm via ribosomes by the activation of intranuclear genes [25]. Thus, poly(P)-trea ted cells might initiate cell-maturation rather than cellular apoptosis or necrosis. Recently, it was reported that poly(P) induces calcium signaling via activation of P2Y receptors, which are purinergic receptors [29]. P2Y is also expressed in osteoblast and associated with maturation of osteoblast [30]. Thus, poly(P) might have played a regulatory role in the osteoblast differentiation via calcium signaling.

In conclusion, MC3T3-E1 cells, which show osteoblastic characteristics at the resting stage, can be induced into the maturation or active phase of osteoblastic cells by poly(P) treatment. Poly(P) could be a good candidate for medical device to regenerate bone tissue.
